# Effects of sea salt intake on metabolites, steroid hormones, and gut microbiota in rats

**DOI:** 10.1371/journal.pone.0269014

**Published:** 2022-08-12

**Authors:** Saoraya Chanmuang, Bo-Min Kim, Su-Yeon Gu, Ye-Jin Son, Huong-Giang Le, Young-Do Nam, Eun-Ji Song, Kyung-Sik Ham, Hyun-Jin Kim

**Affiliations:** 1 Department of Food Science and Technology, and Institute of Agriculture and Life Science, Gyeongsang National University, Jinju, Gyeongsang, Republic of Korea; 2 Division of Applied Life Science (BK21 four), Gyeongsang National University, Jinju, Gyeongsang, Republic of Korea; 3 Research Group of Healthcare, Korea Food Research Institute, Jeollabuk, Republic of Korea; 4 Department of Food Engineering, Mokpo National University, Muan, Jeonnam, Republic of Korea; Tokyo University of Agriculture, JAPAN

## Abstract

High salt intake is positively linked to many health problems, but the effect of mineral-rich sea salt (SS) has rarely been studied. To better understand the physiological effects of SS intake, the changes in general characteristics, metabolites, steroid hormones, and gut microbiota of SS-fed rats were investigated. Male rats were fed either a normal diet (ND, control) or ND containing 1% SS or 4% SS for 5 weeks. SS intake decreased fat, spleen, liver, and body weight, and increased blood urea nitrogen (BUN), water intake, and gut salt content. Accumulated gut salt content led to a decrease in beneficial bacteria, such as *Lachnospiraceae* and *Lactobacillus*, but an increase in potentially harmful bacteria, resulting in a change in lipid metabolites associated with gut health. Interestingly, most renal lysophosphatidylcholines (LPCs) associated with many renal functions were dramatically decreased and female hormones, such as estrogens, were significantly more altered than the male hormones by high SS intake. Although further investigation is needed, these data suggest that high SS intake could be positively linked to kidney dysfunction and gut health problems, and salt-related physiological changes may be sex-specific. Additionally, these data will be useful to better under-stand the physiological effects of SS intake.

## Introduction

Salt, which is mainly composed of sodium and chloride, is essential for human health. Sodium acts as an electrolyte and plays a vital role in the human body by regulating fluid balance, the nervous system, and muscular contraction [1]. On the other hand, excessive salt consumption is linked with many health issues, including cardiovascular diseases, hypertension, osteoporosis, stroke, and kidney dysfunction [2]. Therefore, the World Health Organization (WHO) has recommended reduction in sodium intake to less than 2 g/day (5 g/day salt) in adults to lower their chances of developing such diseases [3]. Nevertheless, researchers have extensively discussed the disadvantages of reducing salt consumption after its association with insulin resistance [4, 5], an increase in plasma cholesterol and triglyceride levels [6], and an increase in premature death [7], were revealed.

Despite many studies regarding the effects of high and low salt intake on human health, the physiological effects of salt consumption remain unclear. Furthermore, the majority of recent studies have focused on specific disorders, such as cardiovascular diseases and hypertension [8, 9], but the overall metabolite outcome in each organ, body fluid, and their associated network has rarely been analyzed. In addition, practically all salt samples used in previous studies were those of purified salt (> 99.95% of NaCl without additional minerals), and only few studies have been carried out about the effects of mineral-rich salt such as sea salt (SS) on health outcomes. SS, which is mainly produced by the evaporation of seawater by wind and sunlight, followed by a ripening process to remove bitterness, contains significant amounts of natural minerals such as Mg^2+^, K^+^, and Ca^2+^ [10]. These minerals have been reported to be beneficial for human health [11]. Mg^2+^, for example, is involved in over 600 enzymatic reactions in the human body [12]; addition of Mg^2+^ and K^+^ to salt has been used to reduce the health risks associated with salt [13].

Therefore, in this study, we investigated the effects of SS intake on general characteristics, general metabolites, steroid hormones, gut microbiota profiles, and the correlation between each parameter in rats to better understand the relationship between mineral-rich SS intake and physiological changes.

## Materials and methods

### Salt and its mineral content

SS was purchased from Taepyungsalt Co., Ltd. (Jeonnam, Korea). NaCl concentrations in salt samples were determined by the Mohr method [14], and their mineral contents were quantitatively analyzed using inductively coupled plasma-optical emission spectrometry (ICP-OES) (Optima 8300, PerkinElmer, Waltham, MA, USA). The water content was determined using MX-50 moisture analyzer (A&D Company, Tokyo, Japan). SS was found to contain 82.55% NaCl and 5.17% water. The content of other trace minerals, namely Mg^2+^, S^2-^, K^+^, and Ca^2+^, was 92.75, 65.01, 32.38, and 15.79 mg/100 g of SS, respectively (S1 Table).

### Animal study

Four-week-old male Sprague Dawley (SD) rats (121.0 ± 5.4 g) were purchased from Koateck (Pyeongtaek, Korea). Rats were housed under controlled environmental conditions with temperatures of 22 ± 2°C, humidity of 55%, and a 12 h light-dark cycle, and were given ad libitum access to food and water. After 1 week of adaptation, rats were randomly allocated into three groups with different diets as follows: normal diet (2018S Teklad Global 18% Protein Rodent Diet, Envigo, IN, US) (control, n = 8), normal diet with 1% SS (SS1%, n = 8), and normal diet with 4% SS (SS4%, n = 8), for 5 weeks. These salt amounts used in this study followed the amount used in previous studies [15]. Body weight of the rats was measured weekly. Food and water intake were monitored daily. Feces samples were collected and put in liquid N_2_ immediately after excretion from the rectum. Urine samples were collected in a metabolic cage containing 0.1% sodium azide for 24 h. After the rats were anesthetized with diethyl ether, plasma was collected from the postcaval vein using VACUETTE^®^ blood collection tubes containing EDTA. The kidney, large intestinal contents (LIC), and whole of the large intestine tissue were collected and immediately frozen in liquid N_2_. All samples were stored at -80°C before analysis. The animal study was approved by the Animal Research Committee of Gyeongsang National University (GNU-151012-R0061).

### Biochemical markers

The following were measured using enzymatic assay kits (Roche Diagnostics, Basel, Switzerland) according to the manufacturer’s instructions: triglyceride (TG), total cholesterol (TC), low-density lipoprotein (LDL) cholesterol, high-density lipoprotein (HDL) cholesterol, aspartate aminotransferase (AST), alanine aminotransferase (ALT), creatinine, and blood urea nitrogen (BUN) in plasma. Additionally, sodium content in LIC was measured using ICP-OES.

### Sample preparation for metabolomic analysis

For metabolite analysis, urine, kidney, and LIC were lyophilized. The urine powder was extracted with water (20 mg/400 μL) containing 8-bromoguanosine (40 μg/mL) as an internal standard using a bullet blender (Next Advance, NY, USA). The kidney powder and LIC were homogenized with 50% acetonitrile (ACN) (25 mg/mL) and 70% methanol (15 mg/700 μL) containing terfenadine, respectively, using a bullet blender. After centrifugation, supernatants were analyzed using ultra-performance liquid chromatography-quadrupole time-of-flight mass spectrometry (UPLC-Q-TOF MS) (Waters, Milford, MA, USA). Additionally, the plasma was mixed with cold acetone (1:1, v/v) containing terfenadine (1 mg/mL) as an internal standard to precipitate the proteins. After centrifugation, the supernatant was analyzed using UPLC-Q-TOF MS.

### UPLC-Q-TOF MS analysis

Metabolite profiles of plasma, urine, kidney, and LIC were analyzed using a UPLC-Q-TOF equipped with an Acquity BEH C18 column (2.1 mm × 100 mm, 1.7 μm; Waters). The samples were injected into the column. The column temperature was set at 40°C; the mobile phase was deionized water containing 0.1% formic acid (A) and ACN containing 0.1% formic acid (B) at a flow rate of 0.35 mL/min. The eluents were detected using Q-TOF MS with positive electrospray ionization (ESI). The desolvation and source temperatures were set at 400°C and 120°C, respectively, and the desolvation gas flow rate was set at 800 L/h. The capillary and sampling cone voltages were set at 3 kV and 40 V, respectively. Leucine-enkephalin ([M+H] = 556.2771) was used as the lock mass of the reference compound at a frequency of 10 s. MS data were obtained with a scan range of 50–1500 m/z with a scan time of 0.2 s. The MS/MS data were obtained in the m/z 50–1500 range using collision energy ramps from 20 to 40 eV. MassLynx^™^ software (Waters) was used for processing of mass spectrometry data, including m/z, retention time, and ion intensity.

### Data processing

Metabolites analyzed by LC/MS data were processed using Markerlynx software for collection, normalization, and alignment. The data peaks were obtained using peak-to-peak baseline noise of 1, noise elimination of 6, peak-width at 5% height of 1s, and an intensity threshold of 50,000. The data were arranged with a 0.05 Da mass window and 0.2 min retention time window. All data were normalized to an internal standard. Metabolites were identified using UNIFI software (Waters) connected to various online databases, ChemSpider (www.chemspider.com), and METLIN database (metlin.scripps.edu).

### Steroid hormone analysis using UPLC-Q-TOF MS

For the analysis of steroid hormones from urine and plasma, the lyophilized urine powder was mixed with 80% methanol (60 mg/200 μL) containing estradiol-d5 as an internal standard and sonicated for 30 min. Plasma steroid hormones were extracted with methanol containing estradiol-d5 for 1 h. After centrifugation, the urine and plasma supernatants were analyzed by UPLC-Q-TOF MS in multiple reaction monitoring (MRM) mode. Precursor and product ions of each steroid hormone were used for the analysis (S2 Table). The column and UPLC-Q-TOF MS analysis conditions were the same as those described above for the analysis of metabolites. Processing of the MRM data was carried out using UNIFI software, which is a data processing tool for mining and sharing of MS data and for identifying metabolites, and all mass data were normalized using internal standards.

### Luteinizing hormone (LH) and follicle stimulating hormone (FSH) analysis

Plasma LH and FSH levels were analyzed using rat luteinizing hormone (LH) and rat follicle-stimulating hormone (FSH) ELISA kits (Cusabio Technology LLC, TX, USA) according to the manufacturer’s instructions.

### Gene expression by real-time PCR analysis

Total RNA was isolated from kidney and colon tissues. To extract total RNA, 30 g of grinded tissue were homogenized using Bloprep-24 homogenizer (Hangzhou Allsheng Instruments Co., Ltd, Hangzhou, China) with 600 μL of the specific buffer provided in the using RNeasy Mini kit (Qiagen, Hilden, Germany). After centrifuging, total RNA was extracted from the supernatant according to the manufacturer’s instructions and its quantity and quality were checked by spectrophotometry. Ten microliters of total RNA from each sample were used to synthesize cDNA using a High-Capacity cDNA Reverse Transcription Kit (ThermoFisher Scientific, MA, US) at 25°C for 10 min, 37°C for 120 min, and 85°C for 5 min. The sample was diluted fifty times before it was stored at -20°C until further processing. Real-time PCR was performed using QuantStudio^™^ 3 Real-Time PCR System (Applied Biosystems, CA, USA) with PowerUp^™^ SYBR^™^ Green Master Mix (ThermoFisher Scientific, MA, USA) and following specific primers: Interleukin-12 (*Il-12*), 5′-ATGATGACCCTGTGCCTTGG-3′ (forward) and 5′-TGCTGCATTTATGGCCTGGA-3’ (reverse); Interleukin 1 (*Il-6*), 5′-TCCTACCCCAACTTCCAATGCTC-3′ (forward) and 5′-TTGGATGGTCTTGGTCCTTAGCC-3′ (reverse); Interleukin 1 beta (*Il-1b*), 5′-TCCTCTGTGACTCGTGGGAT-3′ (forward) and 5′-TCAGACAGCACGAGGCATTT-3′ (reverse); Tumor Necrosis Factor (*Tnf*), 5′-CGTCAGCCGATTTGCCATTT-3′ (forward) and 5′-TCCCTCAGGGGTGTCCTTAG-3′ (reverse); Interferon gamma (*Ifng*), 5′-CGAGGTGAACAACCCACAGA-3′ (forward) and 5′-CGACTCCTTTTCCGCTTCCT-3′ (reverse); Glyceraldehyde 3-phosphate dehydrogenase (*Gapdh*), 5′-GACATGCCGCCTGGAGAAAC-3′ (forward) and 5′-AGCCCAGGATGCCCTTTAGT-3′ (reverse); angiotensin II receptor, type 1a (*Agtr1a*), 5′-CACCAGGTCAAGTGGATTTCG-3′ (forward) and 5′-CTTGGGGCAGTCATCTTGGA-3′ (reverse); Angiotensin-converting enzyme (*Ace*), 5′-GAGGGTCTTTGACGGAAGCA-3′ (forward) and 5′-ATTCGCAGGAACGTGGAACT-3′ (reverse). PCR reaction contained 2.5 μL of cDNA sample, 12.5 μL of SYBR master mix, 5 μL of 1 μM forward primer, and 5 μL of 1 μM reward primer. Molecular grade nuclease-free water was used instead of the sample as a negative control. After an initial denaturation step at 95°C for 10 min, the amplification program consisted of 45 cycles of denaturation at 95°C for 15 s, annealing at 57°C for 30 s, and extension at 72°C for 30 s. Melting curve and gel analyses were used to verify the specific products of the appropriate sizes. Gene expression was assessed by the 2^−ΔΔCt^ method, and gene expression levels were expressed in terms of the relative expression ratio of the target gene to GAPDH for each sample.

### Gut microbiota analysis

The QIAamp DNA Stool Mini Kit (Qiagen) was used to extract bacterial DNA present in the feces of the salt-fed rats. The V1-V2 region of 16S rDNA genes were amplified by polymerase chain reaction using a range of universal primers, viz. 8F (5′-AGAGTTTGATCCTGGCTCAG-3′) and 338R (5′-TGCTGCCTCCCGTAGGAGT-3′), with barcode sequences for multiplexing reads of each sample. DNA library was constructed using Ion Xpress Plus Fragment Library Kit (Thermo Scientific, Wilmington, DE, USA) according to the manufacturer’s instructions, and the final library was quantified using Bioanalyzer 2100 (Agilent Technologies, Inc., Santa Clara, CA, USA) with high-sensitivity DNA chips. Sequencing of the libraries was performed on a 318D sequencing chip using an Ion Sequencing 400 kit and Ion Torrent PGM system (Thermo Scientific) according to the supplier’s instructions. Sequences were processed and analyzed using Quantitative Insights Into Microbial Ecology (QIIME2, version 2020.08) [16]. Amplicon sequence variants (ASVs) were classified within QIIME2 using the SILVA v138 database [17]. Alpha diversity, such as the Chao1 index and Shannon’s diversity, was measured using the qiime diversity alpha command. Bar plot figures for alpha diversity were created using GraphPad Prism v8, with between group statistical differences determined using the Kruskal-Wallis test.

### Statistical analysis

LC/MS data sets were processed by multivariate statistical analysis using SIMCA-P+ version 12.0.1 (Umetrics, Umea, Sweden). Partial least squares discriminant analysis (PLS-DA) score plots were used to visualize differences among samples. In PLS-DA, the goodness of fit measure (R2X and R2Y) and predictive ability (Q2 and R2) were used to evaluate quality, and the permutation test (n = 200) was used to evaluate reliability. Metabolites with a variable importance in the projection (VIP) value > 1.0, were highly relevant for separation among sample groups. Pearson’s correlation coefficients between variables were calculated and visualized using the R software. Heatmaps were visualized using R with the gplots package to evaluate relationships among steroid hormones, metabolites, gut microbiota, and general characteristics. Statistical analysis of all data, including animal characteristics, metabolites, steroid hormones, and gut microbiota, was performed using one-way analysis of variance (ANOVA) with Duncan’s test (p<0.05) using SPSS 23.0 (SPSS Inc., Chicago, IL, USA).

## Results

### Biochemical markers

The general characteristics of the rats fed different diets were investigated (Table 1). Compared with the control and SS1% groups, body and epididymal adipose tissue weights of the SS4% group were significantly reduced by 10% and 30%, respectively, while their food and water intake increased. In addition, the SS4% group showed a decrease in the weight of the liver and spleen, but kidney weight did not change, and the blood urea nitrogen (BUN) values of the SS4% group were 86% higher than those of the other groups. Plasma TG levels decreased in a salt concentration-dependent manner. However, there was no change in plasma TC, LDL, HDL, ALT, AST, and creatinine levels.

**Table 1:** Characteristics of rats fed different concentrations of sea salt.

	Animal models		
	Control	SS 1%	SS 4%
Body weight gain (g)^1^	202.00 ± 16.70^a^	201.13 ± 12.84^a^	180.25 ± 11.39^b^
Food intake (g/day)^1^	38.68 ± 15.68	39.45 ± 16.20	43.69 ± 17.16
Water intake (g/day)^1^	51.08 ± 29.59^b^	58.58 ± 31.85^b^	96.86 ± 47.02^a^
Spleen (g)^1^	0.74 ± 0.06^a^	0.68 ± 0.10^a^	0.60 ± 0.53^b^
Epididymal adipose tissue (g)^1^	3.27 ± 0.37^a^	3.32 ± 0.31^a^	2.29 ± 0.33^b^
Liver (g)^1^	9.86 ± 0.62^a^	9.64 ± 0.79^ab^	9.06 ± 0.70^b^
Kidney (g)^1^	2.05 ± 0.12	2.19 ± 0.13	2.08 ± 0.15
Blood TG (mg/dL)^2^	25.00 ± 2.74^a^	23.60 ± 2.41^ab^	20.40 ± 2.51^b^
Blood TC (mg/dL)^2^	106.80 ± 19.63	107.20 ± 17.43	114.20 ± 8.58
LDL Cholesterol (mg/dL)^2^	53.40 ± 5.22	55.48 ± 9.16	58.12 ± 6.16
HDL Cholesterol (mg/dL)^2^	48.40 ± 14.48	47.00 ± 8.97	52.00 ± 5.96
ALT (unit/L)^2^	27.60 ± 4.04	29.00 ± 4.47	26.80 ± 3.83
AST (unit/L)^2^	65.60 ± 6.66	65.60 ± 5.55	68.20 ± 1.30
Creatinine (mg/dL)^2^	0.28 ± 0.04	0.26 ± 0.05	0.28 ± 0.04
BUN (mg/dL)^2^	18.8 ± 2.82^b^	18.90 ± 1.28^b^	34.92 ± 5.20^a^

TG, triglyceride; TC, total cholesterol; ALT, aspartate aminotransferase; AST, alanine aminotransferase; BUN, blood urea nitrogen. Values were expressed as mean ± SD (^1^ n = 8, ^2^ n = 5) and different letters in the same column indicated significant differences at p-value < 0.05.

### Tissue and biofluid metabolomic analysis from SS-fed rats

**Fig 1:**
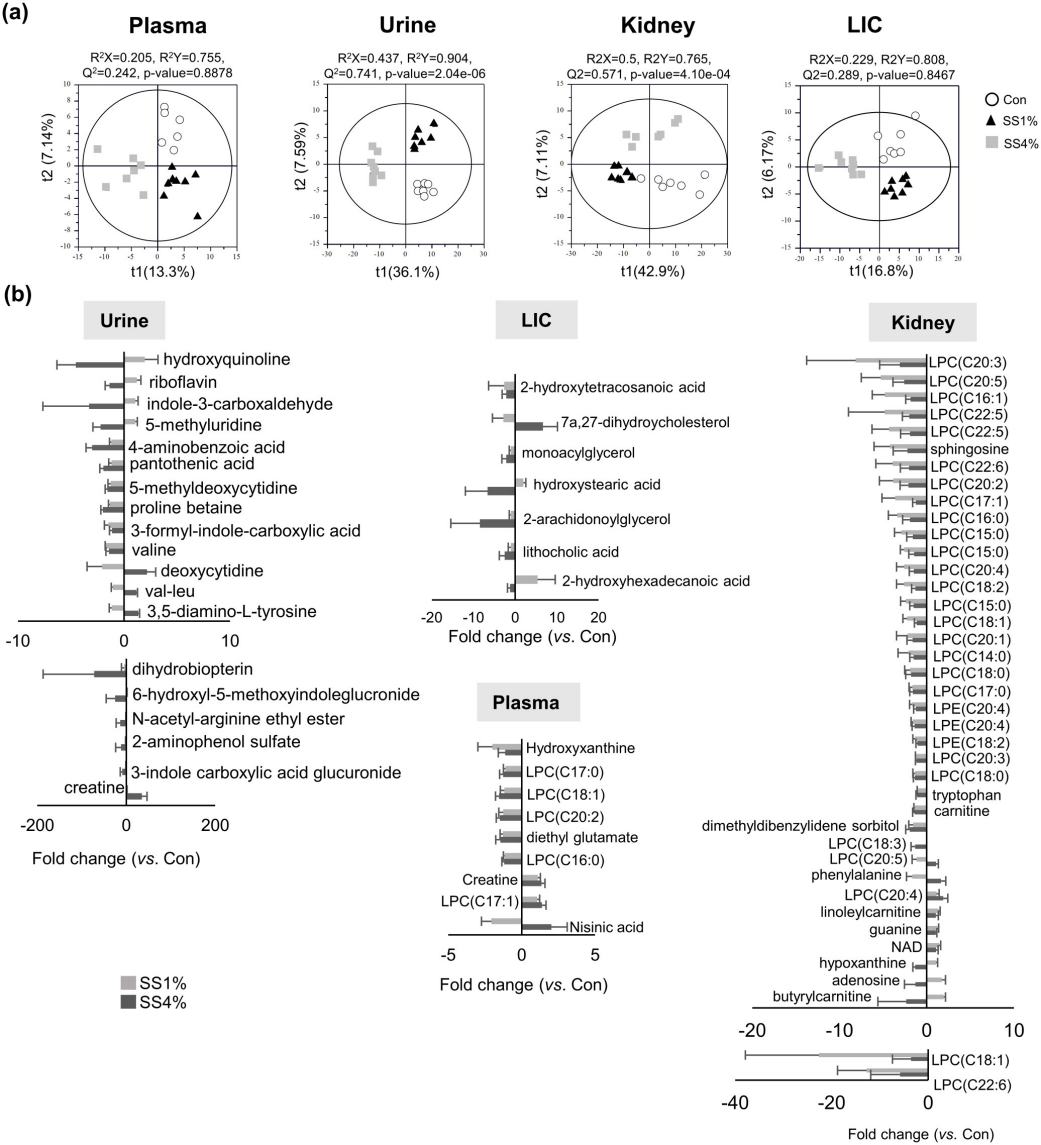
Metabolite analysis of rats fed sea salt at different concentrations. (a) Partial least squares discriminant analysis (PLS-DA) score plots obtained from UPLC-Q-TOF MS data of plasma, urine, kidneys, and large intestinal content (n = 8). (b) Fold change of identified metabolites. Metabolites were analyzed using UPLC-Q-TOF MS equipped the Acquity BEH C18 column (2.1 mm × 100 mm, 1.7 μm) with a positive ESI mode. The qualification of the PLS-DA models was evaluated by R2X, R2Y, Q2, and p-values. R2X and R2Y show the fitting quality of the models, while Q2 shows their prediction quality. Fold changes of SS groups were calculated using normalized chromatogram intensities against control. LIC is large intestine content.

The UPLC-Q-TOF MS data showed that 223 plasma, 247 urine, 295 kidney, and 280 LIC metabolites were detected. Based on these metabolites, the PLS-DA models showed differences among groups, and their quality was evaluated (Fig 1a). Sample groups were significantly distinguished from each other by the first two principal components in the PLS-DA score plots of urine and kidney metabolites with statistically acceptable quality parameters (R2X = 0.437, R2Y = 0.904, Q2 = 0.741, and p-value = 2.04e-06 for urine; R2X = 0.5, R2Y = 0.765, Q2 = 0.571, and p-value = 4.10e-04 for kidney) and good cross-validation (Y-axis of R2 <0.4 and Y-axis of Q2<-0.1). The score plots of plasma and LIC also showed that the sample groups were clearly separated from each other, but the quality parameters and cross-validation of both PLS-DA models were not statistically acceptable (R2X = 0.205, R2Y = 0.755, Q2 = 0.242, and p-value = 0.8878 for plasma; R2X = 0.229, R2Y = 0.808, Q2 = 0.289, and p-value = 0.8467 for LIC). Although the PLS-DA models showed that SS intake had a greater effect on urine and renal metabolite profiles than plasma and LIC, the intensities of all metabolites, including plasma and LIC metabolites, were statistically analyzed. We identified 19 urine, 40 kidney, 9 plasma, and 7 LIC metabolites as the major metabolites contributing to the separation in the score plots (S3 and S4 Tables); the fold changes are shown in Fig 1b (also S5 and S6 Tables). In urine, compared to the control group, the SS4% group showed a significant decrease in almost all identified metabolites except creatine, 3,5-diamino-tyrosine, val-leu, and deoxycytidine. Other metabolites like 4-aminobenzoic acid, proline betaine, 5-methyluridine, indole-3-carboxaldehyde, and hydroxyquinoline decreased more than 2 times. N-acetyl-arginine ethyl ester, 2-aminophenol sulfate, and 3-indole carboxylic acid glucuronide decreased by more than eight times. Moreover, the most reduced metabolites in the SS4% group were 6-hydroxyl-5-methoxyindoleglucronide and dihydrobiopterin, which decreased 25 and 72 times, respectively. In contrast, creatine increased 35 times in the SS4% group. In the kidney, most lysophosphatidylcholines (LPCs) and lysophosphatidylethanolamines (LPEs) dramatically decreased up to 22 times in the SS-fed groups, except for LPC (C20:4). Interestingly, LPCs were observed to have decreased more in the SS1% group than in the SS4% group. LPC (20:5) was observed to have decreased in the SS1% group but increased in the SS4% group. Moreover, compared to the control group, sphingosine and dimethyldibenzylidene sorbitol decreased 4.2- and 1.6-fold in the SS1% group and 2.2- and 1.9-fold in the SS4% groups, respectively; the levels of adenosine and butyrylcarnitine increased 1.7- and 2-fold in the SS1% group, but were found to have decreased 1.3- and 2.4-fold in the SS4% group. In the LIC, monoacylglycerol, 2-arachidonoylglycerol, and lithocholic acid decreased 2.2-, 8.5-, and 2.5-fold in the SS4% group compared to the control group. The 7a,27-dihydroycholesterol level decreased in the SS1% group but increased 6.7-fold in the SS4% group. In contrast, hydroxystearic acid and 2-hydroxyhexadecanoic acid in-creased 1.9- and 5.3-fold in the SS1% group but decreased 6.6- and 1.3-fold in the SS4% group. Plasma metabolites were changed 1.1- to 2-fold by SS intake. Among plasma metabolites, hydroxyxanthine and nisinic acid decrease 2-fold in the SS1% group, whereas nisinic acid increase 2-fold in the SS4% group.

### Steroid hormone profile in the plasma and urine of SS-fed rats

**Fig 2:**
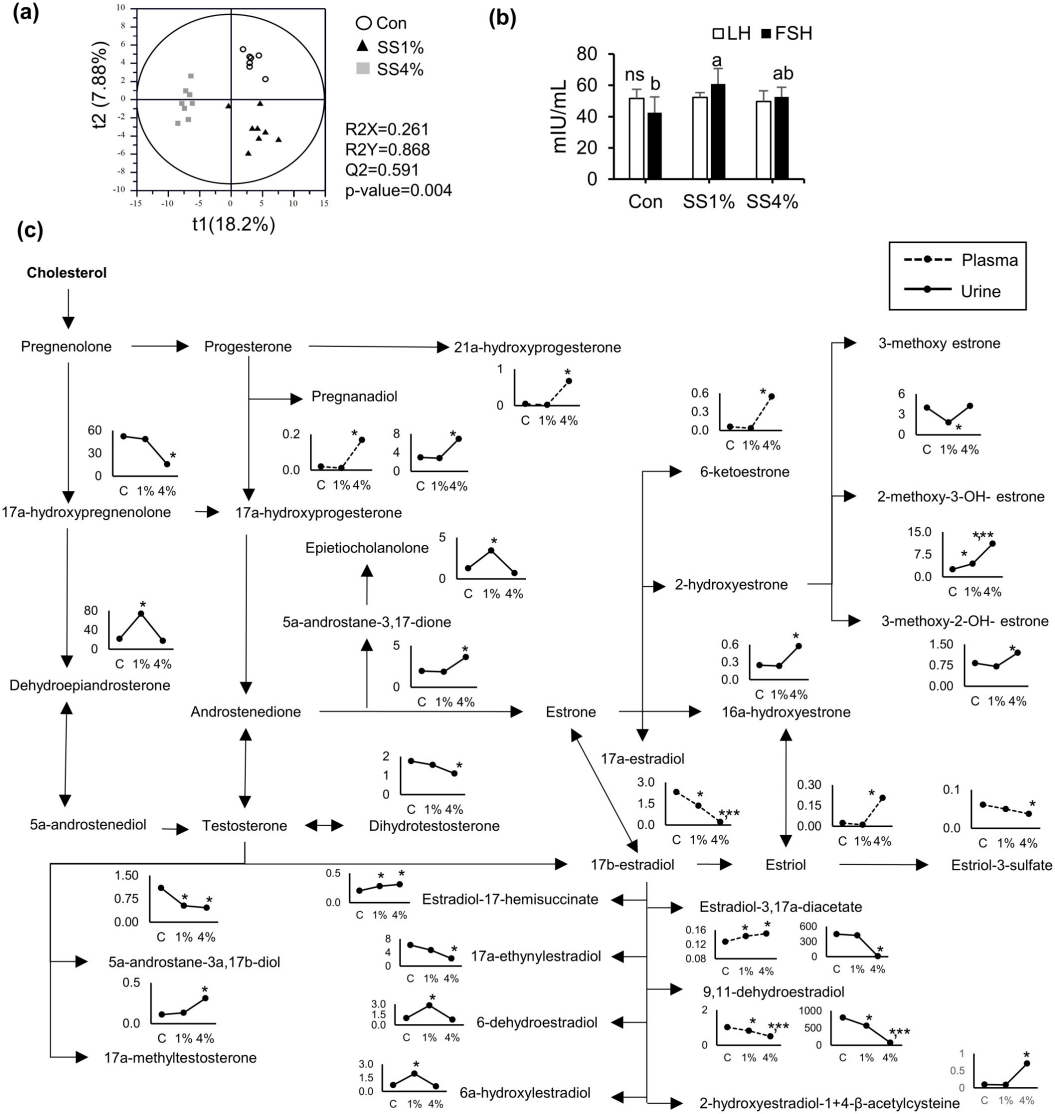
Steroid hormone analysis of plasma and urine samples from rats fed different concentrations of sea salt. (a) Partial least squares discriminant analysis (PLS-DA) score plot. (b) Plasma luteinizing hormone (LH) and follicle-stimulating hormone (FSH) content. (c) Schematic of steroid hormone pathway associated with salt intake and relative abundances of steroid hormones affected by salt intake. Steroid hormones were analyzed using UPLC-Q-TOF MS equipped with the Acquity BEH C18 column (2.1 mm × 100 mm, 1.7 μm) with a positive MRM mode. * p-value < 0.05 vs control and ** p-value < 0.05 vs SS1%. Plasma LH and FSH contents were analyzed by ELISA kit. Data presented as means ± SD (n = 4) and the different letters on the bars indicate significant differences at p-value < 0.05; ns, not significant difference.

Eighty-four plasma and 91 urine steroid hormones were analyzed using UPLC-Q-TOF MS in MRM mode, and the differences among sample groups were visualized using the PLS-DA score plot (Fig 2a). The three groups were significantly separated from each other in the score plot with statistically acceptable quality parameters (R2X = 0.261, R2Y = 0.868, Q2 = 0.591, and p-value = 0.004) and cross-validation values (R2 intercept < 0.5 and Q2 intercept < -0.2). Of these, 27 steroid hormones, including estrone, estradiol, estriol, progesterone, pregneolone, androsterone, and testosterone-based hormones, were identified as the main steroid hormones that contributed to the observed differences among the groups in the score plot (S7 Table). Based on these steroid hormones affected by salt intake, the steroid hormone pathway was proposed and their relative abundances were analyzed (Fig 2c). In urine, estradi-ol-3,17a-diacetate, 9,11-dehydroestradiol, 17a-estradiol, epietiocholanolone, 17a-ethynylestradiol, 17a-hydroxypregenenolone, dehydroepiandrosterone, 5a-androstane-3a,17b-diol, 6-dehydroestradiol, and 6a-hydroxyestradiol in the SS4% group decreased by 119, 52, 23, 5, 4, 4, 4, 3, 3, and 2 times, respectively, whereas most other urinary steroid hormones increased two to seven times. However, the levels of dehydroepiandrosterone, epietiocholanolone, 6-dehydroestradiol, and 6a-hydroxyestradiol, which were increased by 1% SS intake, were returned to control levels after intake of 4% SS to the control levels; on the other hand, 3-methoxy estrone showed an opposite trend. In plasma, 21a-hydroxyprogesterone, pregnanadiol, 6-ketoesterone, and estriol levels in the SS4% group increased 14, 8, 9, and 9 times, respectively, compared to those observed for the control group, whereas 9,11-dehydroestradiol and estriol-3-sulfate decreased 2 times. Moreover, plasma FSH increased by 43% with 1% SS intake, but LH did not change (Fig 2b).

### Gene expression levels of inflammatory-related cytokines in kidney and colon of SS-fed rats

The expression levels of inflammation-related genes, including *Il-12*, *Tnf*, *Ifng*, *Il-6*, and *Il-1b*, in the kidney and colon (S1 Fig) were determined. The results revealed that the expression levels of almost all cytokines were not significantly changed by SS intake. Only the expression level of *Il-6* in the kidney and *Ifnγ* in the colon were significantly increased upon SS intake.

### Gut microbiota

**Fig 3:**
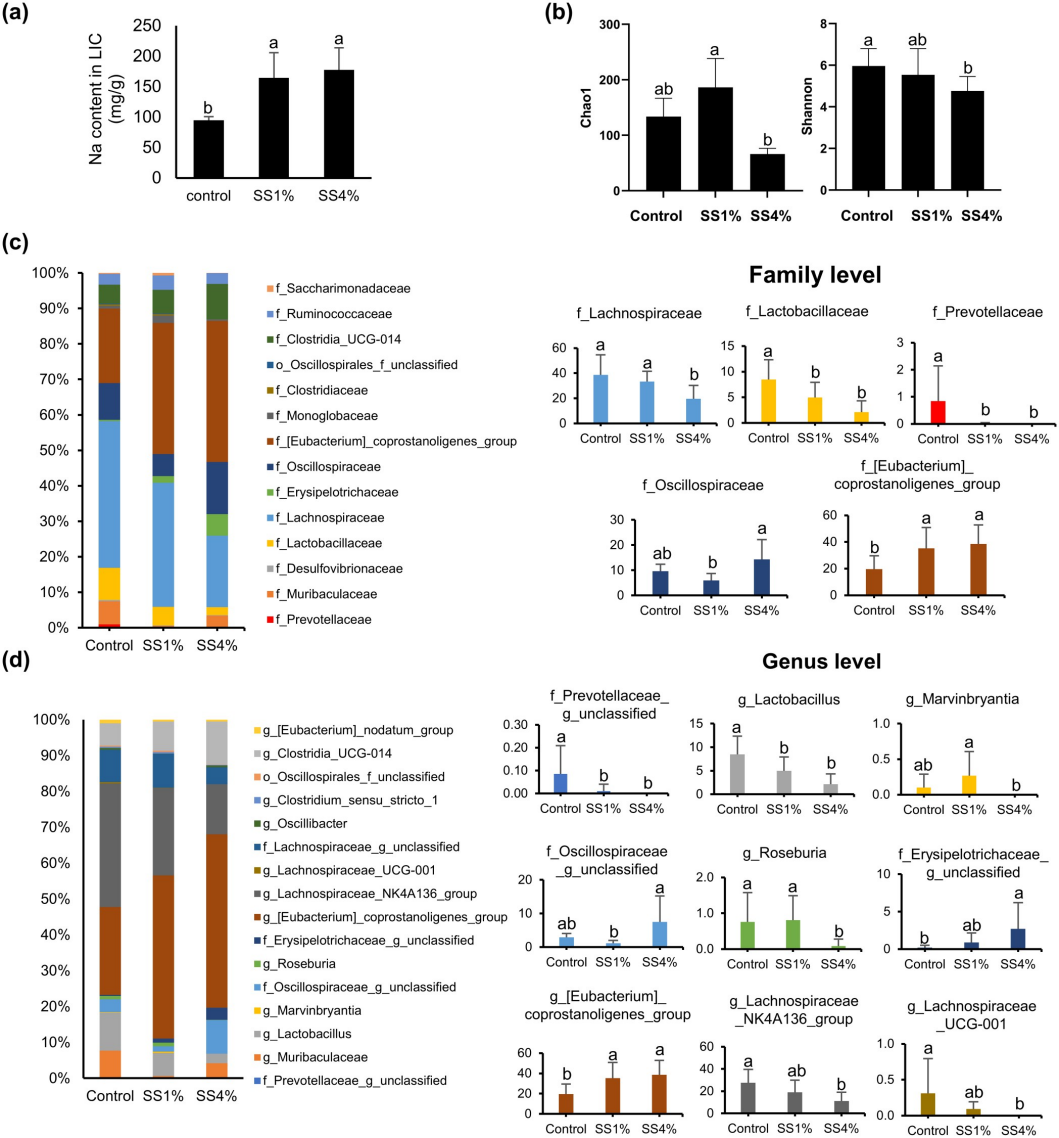
Gut microbiota analysis of feces from rats fed different concentrations of sea salt. (a) The sodium content in large intestinal content (LIC). (b) Chao1 and Shannon indices calculated after rarefying to an equal number of sequence reads for all samples. Bar charts summarizing overall microbial composition in feces of rats fed sea salt diet at family (c) and genus (d) levels with the average relative abundance. QIAamp DNA Stool Mini Kit was used to extract bacterial DNA and the V1-V2 regions of 16S rDNA genes were amplified by polymerase chain reaction. DNA library was constructed and final library was quantified using Bioanalyzer 2100. Sequencing of the libraries was performed on a 318D sequencing chip. Sequences were processed and analyzed using Quantitative Insights Into Microbial Ecology. Amplicon sequence variants were classified within QIIME2 using the SILVA v138 database. Bar graphs present the relative abundance of microbes analyzed from five samples and different letters on the bars indicate significant differences at p-value <0.05.

The sodium content in LIC was significantly increased in the SS1% and SS4% group compared to control group (Fig 3a). The Chao1 and Shannon indices for evaluating the richness and diversity of the gut microbiota of SS-fed rats showed that both were decreased by SS4% intake (Fig 3b). Further, the gut microbiota population of rats was affected by SS intake. At the family level, *Prevotellaceae*, *Lactobacillaceae*, and *Lachnospiraceae* populations of the SS4% group significantly decreased by 49%–100% compared to those of the control group, whereas the population of *Oscillospiraceae* and *Eubacterium* significantly increased by 48%–96% with 4% SS intake (Fig 3c). At the genus level, *Prevotellaceae_g_unclassified*, *Lactobacillus*, *Lachnospiraceae_NK4A136_group*, and *Lachnospiraceae_UCG-001* decreased in a salt concentration-dependent manner. In the SS4% group, *Prevotellaceae_g_unclassified*, *Marvinbryantia*, and *Lachnospiraceae_UCG-001* disappeared, whereas *Roseburia*, and *Lachnospiraceae_NK4A136_group* were reduced by 88% and 59%, respectively. However, unclassified *Oscillospiraceae* and *Erysipelotrichaceae*, and *Eubacterium_coprostanoligenes_group* increased by 2.6, 13, and 2 times, respectively, upon 4% SS intake (Fig 3d).

### Correlation analysis

**Fig 4:**
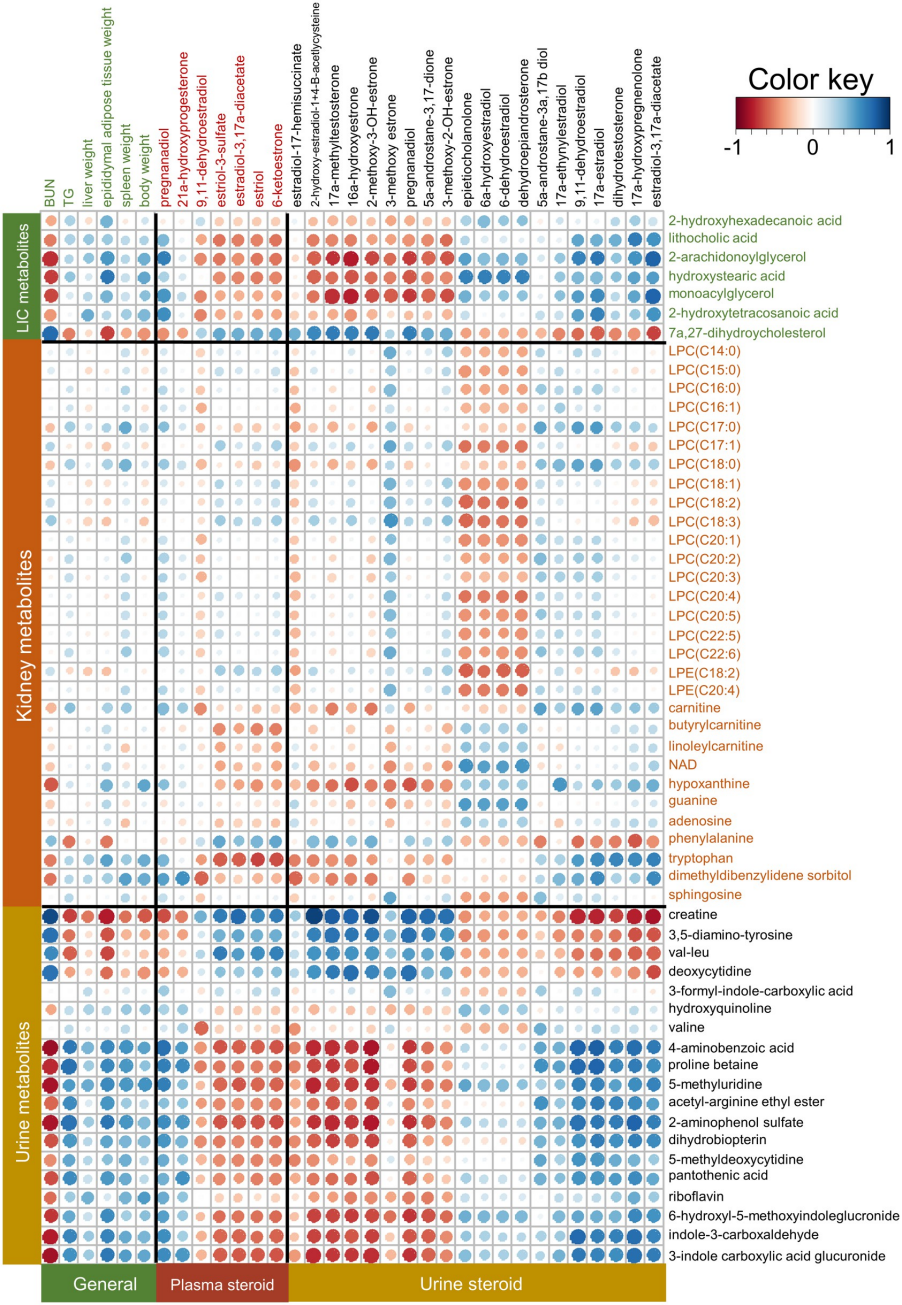
Analysis of correlations between identified metabolites and steroid hormones with general characteristic data. The correlation matrix was analyzed and visualized with a heat map generated with the R corrplot package. Positive correlations are shown in blue and negative correlations are shown in red. A larger circle means a stronger correlation.

The correlations between metabolites, steroid hormones, and general animal characteristics were analyzed (Fig 4). Most urine metabolites, except 3-formyl-indole-carboxylic acid, hydroxyquinoline, and valine, had either positive or negative correlation with steroid hormones and general characteristics. In particular, urine steroid hormones, including estradiol-3,17a-diacetate, 17a-hydroxypregnenolone, dihydrotestosterone, 17a-estradiol, and 9,11-dehydroestradiol, showed moderate to very strong negative correlations (r = -0.82 to -0.41) with urine metabolites, including creatine, 3,5-diamino-tyrosine, val-leu, and deoxycytidine. In contrast, these urine steroid hormones had weak to very strong positive correlations (r = 0.21 to 0.81) with renal tryptophan and urine metabolites, including 4-aminobenzoic acid, proline betaine, 5-methyluridine, acetyl-arginine ethyl ester, 2-aminophenol sulfate, dihydrobiopterin, 5-methyldeoxycytidine, pantothenic acid, 6-hydroxyl-5-methoxyindoleglucronide, indole-3-carboxaldehyde, and 3-indole carboxylic acid glucuronide. A similar correlation was observed between the above urine metabolites, two plasma steroid hormones (21a-hydroxyprogesterone and pregnanadiol), and general characteristics, except BUN. However, the opposite correlation was observed between urine metabolites and urine steroid hormones, including 3-methoxy-2-OH-estrone, 5a-androstane-3,17-dione, pregnanadiol, 2-methoxy-3-OH-estrone, 16a-hydroxyestrone, 17a-methyltestosterone, and 2-hydroxy-estradiol-1+4-β-acetylcysteine, and other plasma steroid hormones. Interestingly, as with these hormones, BUN showed either a strong negative (r = -0.38 to -0.85) or a positive correlation (r = 0.63 to 0.74) with urine metabolites. Most LIC metabolites also had either a negative or a positive correlation with steroid hormones and general characteristics. In particular, 2-arachidonoylglycerol, hydroxystearic acid, and monoacylglycerol in LIC had a positive correlation with estradiol-3,17a-diacetate, 17a-hydroxypregnenolone, 17a-estradiol, 9,11-dehydroestradiol, dehydroepiandrosterone, 6-dehydroestradiol, 6a-hydroxyestradiol, and epietiocholanolone in urine, whereas they showed a negative correlation with most other urine steroid hormones (r = -0.79 to -0.06), most plasma steroid hormones (r = -0.53 to -0.02), and BUN (r = -0.73 to -0.66). However, unlike the above LIC metabolites, 7,27-dihydroycholesterol had an opposite correlation with steroid hormones and general characteristics. In addition, some of the urine steroid hormones, such as dehydroepiandrosterone, 6-dehydroestradiol, 6a-hydroxyestradiol, and epietiocholanolone, showed a negative correlation (r = -0.60 to -0.20) with renal LPCs and LPEs. Moreover, the correlation between gut microbiota and metabolites was also analyzed (S2 Fig). We found that the family *Prevotellaceae* and *Muribaculaceae* had a positive correlation with renal LPCs (r = 0.24 to 0.59) and sphingosine (r = 0.57 to 0.63), but *Eubacterium* showed a negative correlation (r = -0.43 to -0.24). In addition, Oscillospiraceae had strong negative correlations with linoleylcarnitine (r = -0.68) and NAD (r = -0.56) in the kidney. In addition, *Lactobacillaceae* (r = 0.33 to 0.62) and *Lachnospiraceae* (r = 0.25 to 0.40) showed a positive correlation with lithocholic acid, 2-arachidonoylglycerol, monoacylglycerol, and 2-hydroxytetracosanoic acid in LIC, while they had a negative correlation with 7a,27-dihydroycholesterol (r = -0.52 to -039). Furthermore, a correlation between the gut microbiota and steroid hormones was also observed (S3 Fig).

## Discussion

High salt intake is linked to many health problems [2], but the effects of mineral-rich salts such as SS containing Mg^2+^, S^2-^, K^+^, and Ca^2+^ (S1 Table) have rarely been studied. In this study, we investigated effect of SS on the changes in general characteristics, metabolites, steroid hormones, and gut microbiota profiles of rats and evaluated their correlations.

In the general characteristics of SS-fed rats, 4% SS intake significantly reduced fat, spleen, liver, and body weights gain and blood TG levels, while food and water intake were increased (Table 1). This result is similar to the results of studies showing that high salt diet intake led to body weight and fat loss in rodent models (4% and 8% salt) and human study (8.3% salt) [15, 18, 19]; however, opposite results have also been reported indicating that high salt intake (8.5–11.5 g/day) was significantly related to increased body fat mass in both children and adults [20]. Moreover, it is well known that high salt intake increases water intake to control osmotic stress, resulting in kidney damage. BUN level, amount of urea nitrogen in blood that is one of the parameters indicate kidney dysfunction, was increase by SS4%. However, serious kidney damage upon SS intake was not observed, and only a slight increase in kidney damage-related markers, including IL-6 and ACE, was seen unlike many animal studies reporting that a high-salt diet without other minerals stimulated inflammatory cytokines [21]. The gut tissue was also hardly affected by high SS intake. Although no serious damage by SS intake was observed, renal, urinary, and LIC metabolites, plasma and urinary steroid hormones, and gut microbiota were significantly changed by SS intake.

The gut salt content was accumulated upon salt intake, accompanied by a change in the gut microbiota profile. In this study, SS intake significantly reduced *Lachnospiraceae* and *Lactobacillaceae*, which are the main producers of short-chain fatty acids and probiotics, respectively, with various health benefits [22, 23]. Similar results stating that high salt intake reduced the *Lachnospiraceae* family in rodents [24], *Lactobacillus murinus* in mice, and *Lactobacillus spp*. in humans, have also been reported. In particular, decreased Lactobacillus murinus by high salt intake (4% salt) increased Th17 cells, resulting in increased blood pressure [25]. *Prevotellaceae*, known as human gastrointestinal microbiota that helps in the breakdown of protein and carbohydrate foods [26] and is linked with many beneficial as well as harmful effects in the gut [27], was also mostly eliminated by SS intake. In contrast to reduced bacteria, the levels of *Oscillospiraceae* and *Eubacteriaceae* were raised upon SS intake, and similar results have been reported in high salt-fed mice and rats [28]. However, several human studies and a meta-analysis have observed that *Oscillospira* is negatively linked with human leanness and inflammatory diseases [29]. The alteration of gut microbiota profiles upon SS intake revealed that an increase in gut salt content may have a negative effect on gut health. Moreover, changes in the gut microbiota profile may led to changes in gut metabolites, including indoles, bile acids, and fatty acids.

Indole metabolites produced from tryptophan by various gut bacteria were decreased by SS intake may be due to a decrease in Lactobacillus. Among indole metabolites, indole-carboxylic metabolites, which are mainly produced by *Lactobacillus* [30], can circulate systemically, and positively and negatively influence host physiology in the kidney, blood, urine, and gut [31]. In the gut, indole-3-carboxaldehyde was clearly demonstrated to inhibit inflammation and activate increase the immune system through stimulation of IL-22 production by activation of the aryl hydrocarbon receptor in gut lymphoid cells [32]; however, its role in urine remains unclear. Nevertheless, DONALD open cohort study, it was reported that urinary gut bacteria-derived indole-3-carboxaldehyde is negatively associated with elevated C-reactive protein (CRP), a major inflammatory response protein in males during late adolescence-young adulthood [33].

In addition to indoles, bile acids are one of the main metabolites affected by various gut microbiota [34]. In this study, most bile acids, except LCA, were not affected by SS intake. The decreased in LCA, which associated with various gut functions [35], may cause by SS intake reduced LCA production-related microbiota, such as *Lachnospiraceae_bacterium_14–2* and *Lactobacillus* [36]. Along with bile acids, gut lipid metabolites, such as hydroxyl fatty acids and 2-arachidonoylglycerol, which are strongly related with gut health, were also significantly decreased by SS intake. Many of human and animal studies have reported that hydroxyl fatty acids are responsible for its toxic effects, such as inflammation, fever, tissue necrosis, endotoxic shock, and activation of the complement system [37], while 2-arachidonoylglycerol, the major component of the endocannabinoid system in the brain-gut axis [38], reduces metabolic endotoxemia and systemic inflammation [39]. Therefore, this result indicates that the reduction in the levels of these lipid metabolites may have negative effects, including inflammation, on gut health.

Metabolites associated with kidney damage were also found in the kidney and urine samples. The most distinct change observed in the kidneys was the reduction of most LPCs and LPEs (Fig 1b), which have been linked to a variety of physiological and pathological effects [40]. In general, LPCs are considered to be pro-inflammatory and harmful mediators, and are implicated in oxidized LDL-mediated vascular disease [40]; a decrease in LPCs was associated with a risk of adverse outcomes in patients with chronic kidney disease (CKD) and renal failure [41, 42]. It has been clearly shown that LPC has time- and dose-dependent inhibitory effects on acetylcholine-mediated blood vessel vasodilation [43], and impaired vasodilation in the systemic and renal circulation upon high salt intake leading to salt-sensitive hypertension [44].

Moreover, SS consumption reduced urinary dihydrobiopterin levels related to the production of hydrogen peroxide, potentially increasing oxidative stress [45]. A reduction in urinary dihydrobiopterin level may be positively associated with relieving renal oxidative stress. A recent study showed that SS lowered oxidative stress in rats when compared to mineral-deficient salt [10], although it has been previously reported that patients with chronic renal failure had higher dihydrobiopterin levels [46].

In addition to metabolites, many plasma and urinary steroid hormones, which regulate many physiological functions, including reproduction, osmotic regulation, immune system, and gene expression [47, 48], were significantly altered by SS intake.

Although the blood cholesterol profile was not changed by SS intake, the level of pregnenolone, including 17α-hydroxypregnenolone directly synthesized from cholesterol and known as a precursor of most steroid hormones, was decreased upon SS intake. Reduced 17α-hydroxypregnenolone might lead to an alteration in the levels of other steroid hormones. In particular, female hormones, such as estrogens, changed to a greater extent than other steroid hormones. Many human and animal studies have indicated that steroid hormones, including estrogen and aldosterone, play important roles in sodium and water regulation under high salt intake or menopause [47, 49, 50]. In particular, estrogens modulate the function of the kidneys, the main organ regulating sodium reabsorption and water retention via the nitric oxide pathway and angiotensin II system [51]. Estrogen-related sodium retention occurs independently of aldosterone regulation in non-reproductive tissues, such as the kidney tubules. Therefore, estrogen deficiency is strongly associated with blood pressure [47]. The association between blood pressure and steroid hormones clearly demonstrated that aldosterone elevated by estrogen deficiency contributes to increased blood pressure in ovariectomized rats [52] and menopausal women [53]. In this study, many urinary estradiols, the predominant estrogens, were significantly reduced by high SS intake. In particular, the levels of estradiol-3,17a-diacetate and 9,11-dehydroestradiol, the main estradiols, were almost reduced to zero, unlike increased estrones. However, the reduced estradiols hardly increased angiotensin II receptor type 1a (Agtr1a) gene expression, and aldosterone directly related to the blood pressure. Moreover, estradiol has recently been found to increase sodium intake in hypertensive and normotensive female rats [51]. Although further investigation into the correlation between estradiols reduced by SS intake and increased salt consumption is needed, a decrease in estradiol levels may have reduced the desire for more salt intake. Unlike the alteration of female sex hormones, testosterone, the primary sex hormone in males, was not altered by SS intake, except for minor changes in the levels of dihydrotestosterone and 17a-methyltestosterone. However, a different study reported that high-salt (8%)-fed rats showed increased testosterone levels [54]. Although further investigation into the correlation between steroid hormones and physiological changes upon salt intake is needed, these results pertaining to the steroid hormones strongly suggest that salt-related physiological changes may differ with gender because female hormones, such as estrogens, are significantly more affected by SS intake than the male hormones.

Moreover, although the physiological effect of minerals, such as K^+^, Ca^2+^, and Mg^2+^, which are abundant in SS, was not evaluated, and blood pressure was not measured in this study, K^+^, Ca^2+^, and Mg^2+^ have been linked to hypertension in a variety of ways [55]. Human studies have shown that a high K^+^ and Mg^2+^ diet could help to decrease blood pressure [56, 57], and mineral-rich SS was found to cause less hypertension compared to refined salt without minerals in the Dahl salt-sensitive rat [58]. In this study, SS altered metabolite profiles, including reduction of estrogen associated with the regulation of blood pressure [53]. Thus, SS might suppress blood pressure increased by high salt intake through its minerals and the regulation of blood pressure-related metabolites.

## Conclusion

High SS intake altered kidney, urine, and LIC metabolites, steroid hormones, gut microbiota profile, and animal general characteristics; their correlations were also investigated. Although SS-induced serious inflammation in the kidneys and gut was not observed, accumulated gut salt content led to a decrease in beneficial bacteria but an increase in potentially harmful bacteria, resulting in a change in LIC metabolites such as indoles and lipid metabolites. Moreover, the LPCs associated with renal functions dramatically decreased. In particular, female hormones, such as estrogens, which are involved in sodium and water regulation, were significantly more altered by high SS intake than male hormones. In this study, we did not compare the effects of SS and mineral deficient salt and the physiological functions affected by SS intake as well as did not confirm the correlations between metabolites, steroid hormones, and gut microbiota. However, our data suggest that high SS intake could be positively linked to kidney dysfunction and gut health problems, while some observations were not similar to the effects of high consumption of normal salt without minerals. Additionally, steroid hormone data strongly suggest that salt-related physiological changes may be sex-specific. Therefore, these data are useful to better understand the physiological effects of SS intake based on metabolites, steroid hormones, and gut microbiota.

## Supporting information

S1 Table Mineral contents of sea salt (SS). (DOCX)

S2 Table UPLC-Q-TOF MS-MRM conditions for steroid hormone analysis. (DOCX)

S3 Table Identification of plasma and urinary metabolites analyzed by UPLC-Q-TOF MS. (DOCX)

S4 Table Identification of kidney metabolites analyzed by UPLC-Q-TOF MS. (DOCX)

S5 Table Fold changes of plasma, urine, and large intestinal content (LIC) metabolites from rats fed sea salt with different concentrations. (DOCX)

S6 Table Fold changes of kidney metabolites from rats fed sea salt with different concentrations. (DOCX)

S7 Table Plasma and urinary steroid hormone analyzed using UPLC-Q-TOF MS and their fold changes. (DOCX)

S1 Fig Expression levels of inflammatory-related cytokines and hypertension-related genes (Agtr1 and ACE) in kidney. (a) and large intestine tissue (b). Bar graphs present the relative expression of each gene (mean ± SD, n = 5) and different letters on the bars indicate significant differences at p-value <0.05. ns, not significant difference; *Tnf*, tumor necrosis factor alpha; *Ifng*, Interferon gamma; *Ace*, angiotensin converting enzyme; *Agtr1*, Angiotensin II Receptor Type 1.
(TIF)

S2 Fig Analysis of correlations between identified metabolites and gut microbiota. The correlation matrix was analyzed and visualized with a heat map generated with the R corrplot package. Positive correlations are shown in blue, and negative correlations are shown in red.
(TIF)

S3 Fig Analysis of correlations between steroid hormones and gut microbiota. The correlation matrix was analyzed and visualized with a heat map generated with the R corrplot package. Positive correlations are shown in blue, and negative correlations are shown in red.
(TIF)
