# Supplementary material for: Effects of sea salt intake on metabolites, steroid hormones, and gut microbiota in rats
Source: PLoS One. 2022 Aug 12;17(8):e0269014. doi: 10.1371/journal.pone.0269014 (PMC9374251; doi:10.1371/journal.pone.0269014)
Supplement: S1 Table — (DOCX) [file pone.0269014.s001.docx]

**S1 Table.** Mineral contents of sea salt (SS)

| **Mineral** | **Content (mg/100 g of SS)** |
| --- | --- |
| Mg | 92.75 ± 1.36 |
| S | 65.01 ± 3.55 |
| K | 32.38 ± 0.59 |
| Ca | 15.79 ± 0.28 |
| P | 0.15 ± 0.00 |
| Zn | 0.03 ± 0.00 |
| Mn | 0.04 ± 0.00 |
| Cu | 0.00 ± 0.00 |
| Re | 0.12 ± 0.00 |
