# Supplementary material for: Effects of sea salt intake on metabolites, steroid hormones, and gut microbiota in rats
Source: PLoS One. 2022 Aug 12;17(8):e0269014. doi: 10.1371/journal.pone.0269014 (PMC9374251; doi:10.1371/journal.pone.0269014)
Supplement: S2 Table — (DOCX) [file pone.0269014.s002.docx]

**S2 Table.** UPLC-Q-TOF MS-MRM conditions for steroid hormone analysis

| NO | Compounds | MRM | Collision energy (eV) |
| --- | --- | --- | --- |
| 1 | androsterone | 291.2 > 273.2 | 10 |
| 2 | testosterone-17b-glucuronide | 465 > 97 | 20 |
| 3 | epiandrosterone | 273 > 97 | 30 |
| 4 | 21a-hydroxyprogesterone | 331.3 > 313 | 10 |
| 5 | 27-hydroxycholesterol | 367.3 > 114.9 | 20 |
| 6 | allodihydrotestosterone | 291.3 > 159 | 20 |
| 7 | allodihydrotestosterone | 291.3 > 159 | 20 |
| 8 | DHEA | 289.3 > 197 | 20 |
| 9 | estradiol-3-glucuronate | 473.3 > 200.9 | 30 |
| 10 | etiocholanolone | 273 > 91 | 30 |
| 11 | testosterone | 289.2 > 271.2 | 30 |
| 12 | androstenedione | 287 > 97 | 30 |
| 13 | 17a-methyltestosterone | 303 > 97 | 20 |
| 14 | 2-methoxy-3-OH-estradiol | 303.3 > 137.2 | 20 |
| 15 | 17-hydroxyprogesterone | 331.4 > 97.1 | 20 |
| 16 | cortexone | 331.3 > 97.1 | 20 |
| 17 | 6-ketoestradiol-3,17-diacetate | 371.3 > 329.2 | 10 |
| 18 | 17-hydroxyprogesterone | 331.4 > 97.1 | 30 |
| 19 | hydroxyprogesterone caproate | 429.2 > 271.2 | 20 |
| 20 | estiol-16-acetate | 331.3 > 253.1 | 20 |
| 21 | 17-hydroxyprogesterone | 331.4 > 97.1 | 30 |
| 22 | 17-OH-progesterone | 331 > 97 | 30 |
| 23 | ethisterone | 313.4 > 97.1 | 30 |
| 24 | 2,3-dimethoxyestradiol | 317.3 > 302.2 | 10 |
| 25 | dydrogesterone | 313.2 > 295.3 | 10 |
| 26 | cortexolone | 347.3 > 97.1 | 20 |
| 27 | 11-deoxycortisol | 347.3 > 97.1 | 30 |
| 28 | corticosterone | 347.3 > 121 | 20 |
| 29 | equilin-3-SO4 | 347.2 > 267.3 | 20 |
| 30 | 21-deoxycortisol | 347 > 293 | 20 |
| 31 | 21-deoxycortisol | 347.2 > 269.1 | 20 |
| 32 | 21-deoxycortisol | 347.2 > 311.1 | 10 |
| 33 | 9,11-dehydro E2 | 269.3 > 209 | 20 |
| 34 | dehydroepiandrosterone | 289 > 271 | 10 |
| 35 | estriol-16-hemisuccinate | 389.3 > 253.1 | 20 |
| 36 | dihydroequilin-3-SO4 | 373.2 > 332 | 10 |
| 37 | estradiol-3,17a-diacetate | 297.3 > 255.1 | 20 |
| 38 | a-cortol | 333 > 273 | 10 |
| 39 | a-cortol | 333 > 255 | 20 |
| 40 | DHEA-H2O | 253.3 > 197.3 | 20 |
| 41 | 17-hydroxypregnenolone | 333.4 > 297.1 | 20 |
| 42 | 2,3-dimethoxyestrone | 315.3 > 201 | 20 |
| 43 | 17-hydroxypregnenolone | 333.4 > 297.1 | 10 |
| 44 | Progesterone | 315 > 97 | 30 |
| 45 | estriol-16,17-diacetate | 373.3 > 313.2 | 30 |
| NO | Compounds | MRM | Collision energy (eV) |
| 46 | estradiol-17-hemisuccinate | 373.3 > 255.1 | 20 |
| 47 | 17a-hydroxyprogesterone acetate | 373.4 > 313.2 | 20 |
| 48 | estradiol-3,17b-diacetate | 357.3 > 135.2 | 20/30 |
| 49 | medroxyprogesterone acetate | 387.4 > 97.1 | 10 |
| 50 | cortisol | 363.4 > 121.1 | 20 |
| 51 | 17-epiestriol-triacetate | 415.3 > 295.4 | 10 |
| 52 | estriol triacetate | 415.3 > 355.2 | 10 |
| 53 | 16,17-epiestriol triacetate | 415.3 > 295 | 10 |
| 54 | estradiol-3-acetate | 315.3 > 107.1 | 30 |
| 55 | cortisone | 361 > 105 | 30 |
| 56 | 17-methyltestosterone | 303.4 > 91.1 | 30 |
| 57 | pregnenolone | 317.3 > 281.1 | 35 |
| 58 | progesterone | 315.3 > 97.1 | 20 |
| 59 | dydrogesterone | 313.2 > 173.1 | 20 |
| 60 | 3-methoxy-2-OH-estrone | 301.3 > 137 | 20 |
| 61 | epitestosterone | 289 > 97 | 30 |
| 62 | testosterone | 289.3 > 96.9 | 20 |
| 63 | epiandrosterone | 273 > 255 | 20 |
| 64 | androsterone | 273 > 255 | 20 |
| 65 | epiandrosterone | 273 > 255 | 20 |
| 66 | 11a-hydroxy E2 | 289.3 > 253.1 | 20 |
| 67 | 5-androstenediol | 273.3 > 255.1 | 20 |
| 68 | androstenedione | 287.2 > 269.1 | 10 |
| 69 | 2-hydroxyestrone | 287.3 > 268.9 | 30 |
| 70 | 2-hydroxyestriol | 287.3 > 269 | 10 |
| 71 | 3-methoxy estriol | 285.3 > 266.9 | 10 |
| 72 | equilin | 269.3 > 211.1 | 10 |
| 73 | 3-methoxy estriol | 285.3 > 266.9 | 10/20 |
| 74 | 11-ketoestrone | 285.3 > 267.1 | 20 |
| 75 | aldosterone | 361 > 343 | 10 |
| 76 | 6-dehydroestrone | 269.3 > 156.9 | 20 |
| 77 | 9,11-dehydro E1 | 271.3 > 159.3 | 20 |
| 78 | 2-hydroxyestrone | 287.3 > 268.9 | 20 |
| 79 | 11-ketoestrone | 285.3 > 267.1 | 20 |
| 80 | 11-ketoestrone | 285.3 > 267.1 | 20 |
| 81 | 4-hydroxyestrone | 287.3 > 269 | 20 |
| 82 | progesterone | 315 > 109 | 30 |
| 83 | 4-hydroxy-E1-2-glutatione | 592.3 > 317.1 | 10 |
| 84 | pregnenolone | 317.2 > 299.2 | 20 |
| 85 | 2-methoxy-3-OH-estrone | 301.3 > 189.2 | 20 |
| 86 | 4-methoxyestriol | 319.3 > 137.3 | 20 |
| 87 | adrenosterone | 301 > 121.2 | 20 |
| 88 | 4-methoxyestriol | 319.3 > 137.3 | 20 |
| 89 | 2-hydroxy-E1-6-N-3-adenine | 420 > 135.9 | 30 |
