# Supplementary material for: Effects of sea salt intake on metabolites, steroid hormones, and gut microbiota in rats
Source: PLoS One. 2022 Aug 12;17(8):e0269014. doi: 10.1371/journal.pone.0269014 (PMC9374251; doi:10.1371/journal.pone.0269014)
Supplement: S3 Table — (DOCX) [file pone.0269014.s003.docx]

**S3 Table.** Identification of plasma and urinary metabolites analyzed by UPLC-Q­TOF MS

| **No.** | **RT (min)** | **Compound** | **Exact mass** | **MS Fragments** | **VIP** | ***p*-value** |
| --- | --- | --- | --- | --- | --- | --- |
|  |  |  | **(M+H)** |  |  |  |
|  | 0.76 | Creatine | 132.0778 | 90 | 1.54 | 1.71E-02 |
|  | 1.03 | Diethyl glutamate | 204.1241 | 112, 141 | 1.61 | 1.35E-02 |
|  | 1.03 | Hydroxyxanthine | 169.0367 | 112, 141, 152 | 1.57 | 4.35E-02 |
| Plasma | 5.71 | Nisinic acid | 357.2792 | 311, 339 | 1.64 | 1.88E-03 |
|  | 7.26 | LPC(C16:0) 2M | 991.6712 | 104, 184, 496 | 1.44 | 4.35E-02 |
|  | 7.60 | LPC(C20:2) | 548.3709 | 104, 184 | 1.59 | 9.64E-03 |
|  | 7.63 | LPC(C17:1) | 508.3738 | 104, 184 | 1.42 | 1.30E-02 |
|  | 7.66 | LPC(C17:0) | 510.3555 | 104, 184 | 1.79 | 8.32E-04 |
|  | 7.88 | LPC(C18:1) | 524.3710 | 104, 184 | 1.28 | 3.64E-02 |
|  | 0.68 | valine | 118.0874 | 72 | 1.66 | 1.40E-02 |
|  | 0.73 | 4-aminobenzoic acid | 138.0563 | 92, 94, 121 | 1.19 | 1.79E-08 |
|  | 0.73 | creatine | 132.0780 | 90 | 1.17 | 9.58E-10 |
|  | 0.79 | prolin betaine | 144.1031 | 98, 126 | 1.51 | 6.47E-09 |
|  | 0.93 | 5-methyluridine | 259.0933 | 127 | 1.08 | 6.05E-06 |
|  | 0.96 | 3,5-diamino-L-tyrosine | 212.1042 | 109, 168, 194 | 1.12 | 1.41E-05 |
|  | 1.00 | val-leu | 231.1713 | 84, 130 | 1.02 | 5.37E-04 |
|  | 1.20 | N-acetyl-arginine ethyl ester | 245.1509 | 71, 158, 172 | 1.57 | 5.67E-06 |
|  | 1.28 | deoxycytidine | 455.1887 | 95, 112 | 1.19 | 1.06E-04 |
| Urine | 1.90 | 2-aminophenol sulfate | 190.0182 | 110, 172 | 1.17 | 3.17E-08 |
|  | 1.92 | dihydrobiopterin | 240.1109 | 164, 222 | 1.41 | 1.12E-05 |
|  | 2.22 | 5-methyldeoxycytidine 2M | 483.2199 | 109, 126, 242 | 1.20 | 3.50E-03 |
|  | 4.04 | pantothenic acid | 220.1192 | 88, 202 | 1.10 | 1.01E-04 |
|  | 5.07 | 3-formyl-indole-carboxylic acid | 190.0511 | 116, 144, 162 | 1.55 | 1.04E-02 |
|  | 5.20 | indole-3-carboxaldehyde | 146.0614 | 116 | 1.07 | 3.99E-06 |
|  | 5.86 | 3-indole carboxylic acid glucuronide | 338.0879 | 144, 162 | 1.15 | 3.63E-08 |
|  | 6.48 | riboflavin | 377.1457 | 243, 359 | 0.97 | 3.48E-02 |
|  | 6.53 | 6-hydroxyl-5-methoxyindoleglucronide 2M | 679.1992 | 340, 164 | 1.28 | 5.76E-06 |
|  | 7.42 | hydroxyquinoline | 146.0613 | 128 | 1.18 | 2.75E-02 |
