# Supplementary material for: Effects of sea salt intake on metabolites, steroid hormones, and gut microbiota in rats
Source: PLoS One. 2022 Aug 12;17(8):e0269014. doi: 10.1371/journal.pone.0269014 (PMC9374251; doi:10.1371/journal.pone.0269014)
Supplement: S4 Table — (DOCX) [file pone.0269014.s004.docx]

**S4 Table.** Identification of kidney metabolites analyzed by UPLC-Q­TOF MS

| **RT (min)** | **Compound** | **Exact mass** | **MS Fragments** | **VIP** | ***p*-value** |
| --- | --- | --- | --- | --- | --- |
|  |  | **(M+H)** |  |  |  |
| 0.70 | carnitine | 162.1135 | 85, 103 | 1.62 | 6.01E-04 |
| 0.95 | NAD | 664.1161 | 428, 524 | 1.10 | 6.21E-04 |
| 0.95 | hypoxanthine | 137.0470 | 94, 119 | 1.78 | 1.52E-02 |
| 2.53 | guanine | 152.0578 | 110, 152 | 0.93 | 4.67E-03 |
| 2.53 | adenosine | 268.1045 | 136 | 0.76 | 5.07E-02 |
| 2.71 | phenylalanine | 166.0874 | 120 | 1.57 | 2.96E-02 |
| 2.91 | butyrylcarnitine | 232.1553 | 85 | 0.86 | 1.60E-02 |
| 3.00 | Tryptophan | 205.0977 | 188, 146 | 1.89 | 1.63E-03 |
| 6.25 | dimethyldibenzylidene sorbitol | 415.2113 | 119, 281 | 1.86 | 1.59E-02 |
| 6.51 | LPC(C14:0) | 468.3077 | 104, 184 | 0.77 | 3.01E-04 |
| 6.54 | LPC(C20:5) | 542.3235 | 104, 184 | 1.17 | 4.10E-02 |
| 6.55 | linoleylcarnitine | 424.3423 | 85, 365 | 0.82 | 1.55E-06 |
| 6.56 | LPC(C18:3) | 518.3235 | 104, 184 | 1.28 | 3.07E-03 |
| 6.69 | LPC(C16:1) | 494.3240 | 104, 184 | 0.93 | 2.27E-04 |
| 6.79 | LPE(C20:4) | 502.2932 | 119, 287, 361 | 1.15 | 1.76E-04 |
| 6.86 | LPC(C22:6) | 568.3399 | 104, 184 | 1.08 | 1.59E-04 |
| 6.89 | LPC(C15:0) | 482.3226 | 104, 184 | 1.02 | 8.90E-05 |
| 6.90 | LPE(C20:4) | 502.2934 | 119, 287, 361 | 1.02 | 8.05E-03 |
| 6.91 | LPE(C18:2) | 478.2930 | 337 | 0.94 | 8.68E-05 |
| 6.93 | LPC(C20:4) | 544.3402 | 104, 184 | 1.02 | 4.70E-04 |
| 6.94 | LPC(C18:2) | 520.3405 | 104, 184 | 1.00 | 3.94E-02 |
| 6.95 | LPC(C20:5) | 542.3212 | 104, 184 | 1.06 | 3.16E-04 |
| 7.06 | LPC(C22:5) | 570.3553 | 104, 184 | 0.98 | 1.55E-03 |
| 7.19 | LPC(C22:6) | 568.3376 | 104, 184 | 1.15 | 6.55E-04 |
| 7.19 | LPC(C20:3) | 546.3550 | 104, 184 | 1.07 | 4.37E-04 |
| 7.26 | LPC(C22:5) | 570.3545 | 104, 184 | 1.07 | 3.54E-04 |
| 7.27 | LPC(C16:0) | 991.6714 | 104, 184, 496 | 1.05 | 2.52E-04 |
| 7.30 | LPC(C18:1) | 522.3554 | 104, 184 | 1.13 | 1.54E-02 |
| 7.42 | LPC(C20:4) | 544.3371 | 104, 184 | 1.89 | 1.54E-02 |
| 7.43 | LPC(C18:1) | 1043.703 | 104, 184, 522 | 0.92 | 1.16E-03 |
| 7.60 | LPC(C20:2) | 548.3711 | 104, 184 | 1.13 | 1.83E-04 |
| 7.63 | LPC(C17:1) | 508.3753 | 104, 184 | 1.14 | 9.39E-05 |
| 7.66 | LPC(C17:0) | 510.3555 | 104, 184 | 1.46 | 7.96E-04 |
| 7.87 | LPC(C15:0) | 482.3242 | 104, 184 | 0.86 | 3.80E-03 |
| 7.89 | LPC(C18:0) | 524.3713 | 104, 184 | 1.52 | 3.42E-03 |
| 8.03 | LPC(C15:0) | 482.3243 | 104, 184 | 0.89 | 2.57E-03 |
| 8.04 | LPC(C18:0) | 1047.7340 | 104, 184, 524 | 1.37 | 1.94E-03 |
| 8.05 | LPC(C20:3) | 546.3529 | 104, 184 | 1.32 | 1.71E-03 |
| 8.13 | LPC(C20:1) | 550.3858 | 104, 184 | 0.99 | 4.19E-04 |
| 8.17 | sphingosine | 300.2905 | 282, 283 | 0.96 | 1.09E-03 |
