# Supplementary material for: Effects of sea salt intake on metabolites, steroid hormones, and gut microbiota in rats
Source: PLoS One. 2022 Aug 12;17(8):e0269014. doi: 10.1371/journal.pone.0269014 (PMC9374251; doi:10.1371/journal.pone.0269014)
Supplement: S5 Table — (DOCX) [file pone.0269014.s005.docx]

**S5 Table.** Fold changes of plasma, urine, and large intestinal content (LIC) metabolites from rats fed sea salt with different concentrations

|  | Metabolite | Fold change (vs. Control) | |
| --- | --- | --- | --- |
|  |  | SS 1% | SS 4% |
|  | Creatine | -1.10 | +1.35 |
|  | Diethyl glutamate | -1.28 | -1.46 |
|  | Hydroxyxanthine | -2.00 | -1.14 |
| Plasma | Nisinic acid | -2.06 | +2.02 |
|  | LPC(C16:0) | -1.17 | -1.20 |
|  | LPC(C20:2) | -1.27 | -1.49 |
|  | LPC(C17:1) | +1.05 | +1.37 |
|  | LPC(C17:0) | -1.19 | -1.55 |
|  | LPC(C18:1) | -1.11 | -1.28 |
|  | Valine | -1.74 | -1.41 |
|  | 4-aminobenzoic acid | -1.20 | -3.00 |
|  | Creatine | +1.37 | +34.88 |
|  | prolin betaine | -1.31 | -2.00 |
|  | 5-methyluridine | +1.06 | -2.21 |
|  | 3,5-diamino-L-tyrosine | -1.15 | +1.36 |
|  | val-leu | -1.06 | +1.18 |
| Urine | N-acetyl-arginine ethyl ester | -1.21 | -13.49 |
|  | Deoxycytidine | -2.07 | +2.18 |
|  | 2-aminophenol sulfate | -1.27 | -12.08 |
|  | Dihydrobiopterin | -5.06 | -72.51 |
|  | 5-methyldeoxycytidine | -1.25 | -1.56 |
|  | pantothenic acid | -1.18 | -1.96 |
|  | 3-formyl-indole-carboxylic acid | -1.46 | -1.14 |
|  | indole-3-carboxaldehyde | +1.07 | -3.30 |
|  | 3-indole carboxylic acid glucuronide | -1.01 | -8.94 |
|  | Riboflavin | +1.21 | -1.36 |
|  | 6-hydroxyl-5-methoxyindoleglucronide | +1.42 | -25.26 |
|  | Hydroxyquinoline | +1.95 | -4.56 |
|  | 2-hydroxyhexadecanoic acid | +5.37 | -1.25 |
|  | lithocholic acid | -1.04 | -2.51 |
|  | 2-arachidonoylglycerol | -1.04 | -8.48 |
| LIC | hydroxystearic acid | +1.93 | -6.61 |
|  | monoacylglycerol | -1.05 | -2.16 |
|  | 7a,27-dihydroycholesterol | -2.87 | +6.67 |
|  | 2-hydroxytetracosanoic acid | -2.66 | -2.16 |
