# Supplementary material for: Effects of sea salt intake on metabolites, steroid hormones, and gut microbiota in rats
Source: PLoS One. 2022 Aug 12;17(8):e0269014. doi: 10.1371/journal.pone.0269014 (PMC9374251; doi:10.1371/journal.pone.0269014)
Supplement: S6 Table — (DOCX) [file pone.0269014.s006.docx]

**S6 Table.** Fold changes of kidney metabolites from rats fed sea salt with different concentrations

| Metabolite | Fold change (vs. Control) | |
| --- | --- | --- |
|  | SS 1% | SS 4% |
| carnitine | -1.36 | -1.40 |
| NAD | +1.42 | +1.06 |
| hypoxanthine | +1.07 | -1.32 |
| guanine | +1.24 | +1.05 |
| adenosine | +1.72 | -1.30 |
| phenylalanine | -1.64 | +1.62 |
| butyrylcarnitine | +1.95 | -2.35 |
| tryptophan | -1.03 | -1.13 |
| dimethyldibenzylidene sorbitol | -1.58 | -1.94 |
| LPC(C14:0) | -2.08 | -1.50 |
| LPC(C20:5) | -5.30 | -2.58 |
| linoleylcarnitine | +1.33 | +1.05 |
| LPC(C18:3) | 0 | -1.31 |
| LPC(C16:1) | -4.82 | -1.86 |
| LPE(C20:4) | -1.63 | -1.38 |
| LPC(C22:6) | -3.91 | -2.02 |
| LPC(C15:0) | -2.38 | -1.49 |
| LPE(C20:4) | -1.68 | -1.25 |
| LPE(C18:2) | -1.35 | -1.06 |
| LPC(C20:4) | -2.58 | -1.49 |
| LPC(C18:2) | -2.59 | -1.30 |
| LPC(C20:5) | -1.14 | +1.07 |
| LPC(C22:5) | -4.32 | -1.91 |
| LPC(C22:6) | -12.75 | -5.84 |
| LPC(C20:3) | -8.15 | -3.09 |
| LPC(C22:5) | -4.83 | -1.99 |
| LPC(C16:0) | -3.39 | -1.91 |
| LPC(C18:1) | -2.24 | -1.15 |
| LPC(C20:4) | +1.13 | +1.88 |
| LPC(C18:1) | -22.63 | -3.55 |
| LPC(C20:2) | -3.85 | -2.10 |
| LPC(C17:1) | -3.60 | -1.25 |
| LPC(C17:0) | -1.65 | -1.60 |
| LPC(C15:0) | -2.90 | -1.59 |
| LPC(C18:0) | -1.34 | -1.35 |
| LPC(C15:0) | -2.58 | -1.46 |
| LPC(C18:0) | -1.91 | -1.79 |
| LPC(C20:3) | -1.21 | -1.17 |
| LPC(C20:1) | -2.21 | -1.63 |
| sphingosine | -4.19 | -2.22 |
