# Supplementary material for: Effects of sea salt intake on metabolites, steroid hormones, and gut microbiota in rats
Source: PLoS One. 2022 Aug 12;17(8):e0269014. doi: 10.1371/journal.pone.0269014 (PMC9374251; doi:10.1371/journal.pone.0269014)
Supplement: S7 Table — (DOCX) [file pone.0269014.s007.docx]

**S7 Table.** Plasma and urinary steroid hormone analyzed using UPLC-Q-TOF MS and their fold changes

| Sample | Steroid hormones | Analyte MRM | *p*-value^b^ | VIP^c^ | Fold change (vs control) | |
| --- | --- | --- | --- | --- | --- | --- |
|  |  |  |  |  | SS 1% | SS 4% |
| Plasma | Pregnanadiol | 285.1 > 189.0 | 1.82e-3 | 1.60 | -2.59 | +8.52 |
|  | 21a-hydroxyprogesterone | 331.3 > 313.0 | 5.13e-3 | 1.50 | -3.14 | +14.34 |
|  | 6-ketoestrone | 285.3 > 133.0 | 1.43e-3 | 1.62 | -2.95 | +9.34 |
|  | Estradiol-3,17a-diacetate | 297.3 > 255.1 | 1.44e-2 | 1.62 | +1.12 | +1.17 |
|  | 9,11-dehydroestrdiol | 269.3 > 209.0 | 1.22e-3 | 1.71 | -1.33 | -2.17 |
|  | Estriol | 271.2 > 253.2 | 2.91e-3 | 1.56 | -4.84 | +8.72 |
|  | Estriol-3-sulfate | 367.2 > 287.3 | 2.20e-2 | 1.42 | -1.26 | -1.82 |
|  | Epietiocholanolone | 273.1 > 147.0 | 1.45e-3 | 1.56 | +2.66 | -5.32 |
|  | 17a-hydroxypregenenolone | 348.2 > 330.1 | 1.91e-5 | 1.38 | -1.17 | -4.19 |
|  | Pregnanadiol | 285.1 > 189.0 | 3.91e-6 | 1.40 | -1.22 | +2.34 |
|  | Dehydroepiandrosterone | 271.2 > 253.2 | 3.19e-3 | 1.59 | +3.42 | -3.52 |
|  | 5a-androstane-3a,17b-diol | 275.0 >257.0 | 4.10e-3 | 1.68 | -2.45 | -2.74 |
|  | 5a-androstane-3,17-dione | 289.0 > 271.0 | 2.83e-3 | 1.11 | -1.34 | +1.86 |
|  | Dihydrotestosterone | 291.3 > 255.4 | 1.05e-3 | 1.33 | -1.16 | -1.68 |
|  | 17a-methyltestosterone | 303.3 > 97.0 | 4.18e-7 | 1.48 | +1.19 | +2.78 |
| Urine | 2-methoxy-3-OH-estrone | 301.3 > 189.2 | 5.70e-8 | 1.58 | +1.75 | +4.40 |
|  | 3-methoxy-2-OH-estrone | 301.3 > 137.0 | 3.83e-3 | 1.13 | -1.26 | +1.45 |
|  | 3-methoxy estrone | 285.1 > 147.1 | 5.12e-3 | 1.52 | -2.59 | +1.06 |
|  | 16a-hydroxyestrone | 285.0 > 145.0 | 1.38e-6 | 1.43 | -1.27 | +2.31 |
|  | Estradiol-3,17a-diacetate | 297.1 > 255.1 | 4.98e-7 | 1.47 | -1.27 | -119.35 |
|  | 17a-estradiol | 255.1 > 159.1 | 1.44e-8 | 1.83 | -1.94 | -23.39 |
|  | 9,11-dehydroestradiol | 269.1 > 209.0 | 6.53e-6 | 1.55 | -1.93 | -52.34 |
|  | 17a-ethynylestradiol | 279.1 > 133.1 | 1.75e-2 | 1.10 | -1.48 | -4.17 |
|  | 6-dehydroestradiol | 271.2 > 137.2 | 3.57e-3 | 1.56 | +2.83 | -2.93 |
|  | 6a-hydroxyestradiol | 271.2 > 156.9 | 2.68e-3 | 1.58 | +2.74 | -2.41 |
|  | estradiol-17-hemisuccinate | 373.3 > 255.1 | 3.24e-2 | 1.30 | +1.40 | +1.56 |
|  | 2-hydroxyestradiol-1+4-β-acetylcysteine | 408.3 > 319.0 | 3.08e-5 | 1.34 | -1.41 | +6.97 |
