# Supplementary figures and images for: Effects of sea salt intake on metabolites, steroid hormones, and gut microbiota in rats
Source: PLoS One. 2022 Aug 12;17(8):e0269014. doi: 10.1371/journal.pone.0269014 (PMC9374251; doi:10.1371/journal.pone.0269014)

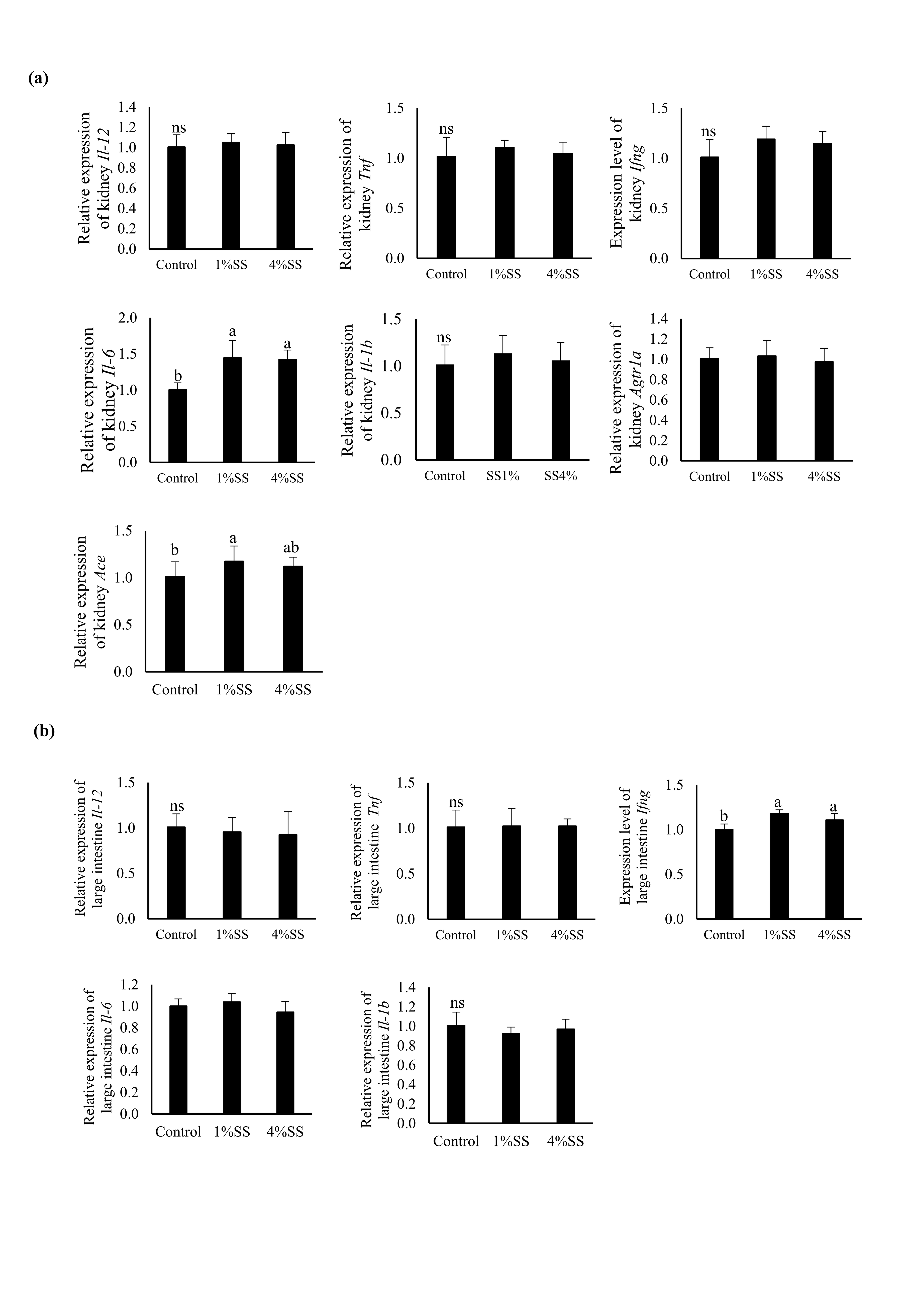

Supplement: S1 Fig — (a) and large intestine tissue (b). Bar graphs present the relative expression of each gene (mean ± SD, n = 5) and different letters on the bars indicate significant differences at p-value <0.05. ns, not significant difference; Tnf, tumor necrosis factor alpha; Ifng, Interferon gamma; Ace, angiotensin converting enzyme; Agtr1, Angiotensin II Receptor Type 1. (TIF) [file pone.0269014.s008.tif]

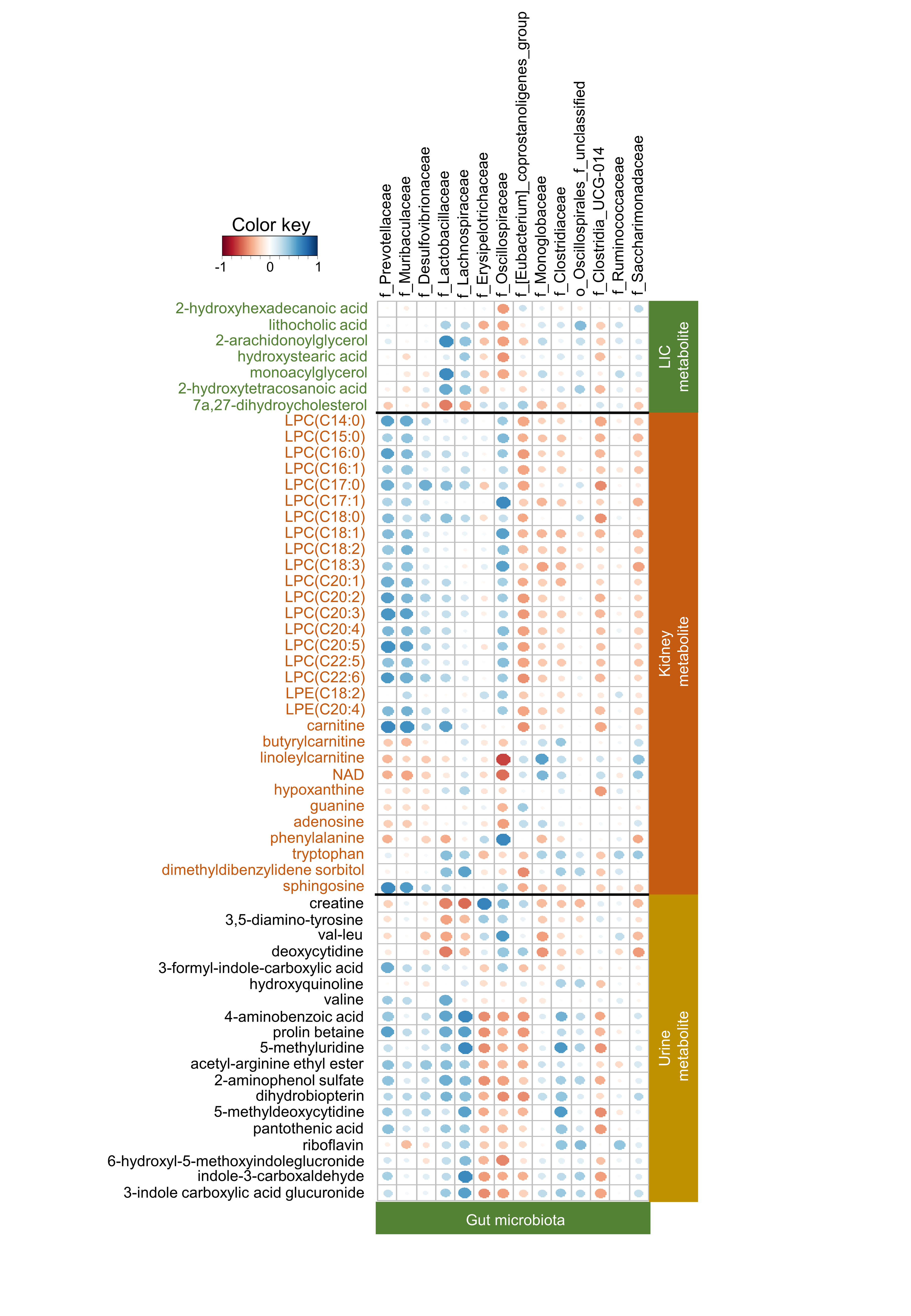

Supplement: S2 Fig — The correlation matrix was analyzed and visualized with a heat map generated with the R corrplot package. Positive correlations are shown in blue, and negative correlations are shown in red. (TIF) [file pone.0269014.s009.tif]

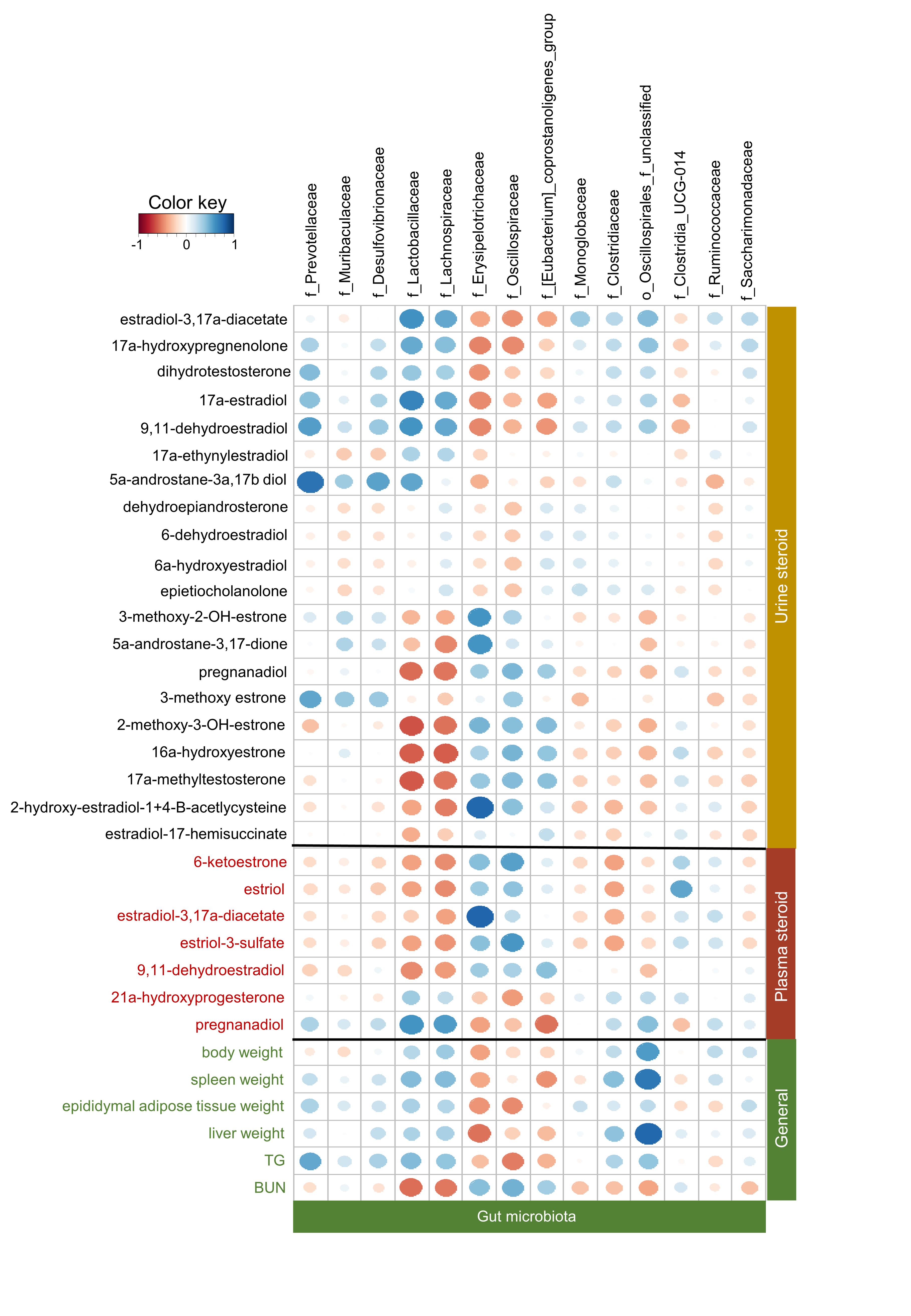

Supplement: S3 Fig — The correlation matrix was analyzed and visualized with a heat map generated with the R corrplot package. Positive correlations are shown in blue, and negative correlations are shown in red. (TIF) [file pone.0269014.s010.tif]
